# The Path to Exhaustion: Time-Variability Properties of Coordinative Variables during Continuous Exercise

**DOI:** 10.3389/fphys.2016.00037

**Published:** 2016-02-15

**Authors:** Pablo Vázquez, Robert Hristovski, Natàlia Balagué

**Affiliations:** ^1^Institut Nacional d'Educació Física de Catalunya, Complex Systems in Sport Research Group, Universitat de BarcelonaBarcelona, Spain; ^2^Ss. Cyril and Methodius, Faculty of Physical Education, Sport and Health, Complex Systems in Sport Research GroupSkopje, Macedonia

**Keywords:** anti-persistent fBm, persistent fBm, synergy, fatigue, exercise, detrended fluctuation analysis

## Abstract

The aim of this study was to detect qualitative changes in the structure of coordinative variable (elbow angle) fluctuations during a quasi-isometric exercise performed until exhaustion. Seven physical education students performed a quasi-isometric arm-curl exercise holding an Olympic bar (weight: 80% 1RM) with an initial elbow flexion of 90° three times over a period of 4 weeks. They were encouraged to persist, even if the elbow angle was lost, until the fatigue-induced spontaneous termination point (FISTP). Changes in both elbow angles were registered during the task through an electrogoniometer. Detrended Fluctuation Analysis (DFA) was conducted on the initial and final 1024 data points of the series and the associated Hurst exponents were obtained. Multi-way RM ANOVA analyses revealed a significant main effect of the Time on task on the Hurst exponent values but also revealed a significant Trial × Time on task interaction. In the initial (non-fatigue) condition participants tended to produce anti-persistent fBm fluctuations. In the final part before exhaustion a tendency toward persistent fBm was dominant. The trial to trial differences in time-variability structure points to an existence of a long-term variability in control strategies during exercise. The changes in the temporal structure of the elbow angle variability as effort accumulated reflected an increase in low-frequency fluctuations signifying a change in psychobiological mechanisms used to negotiate the task demands. The variability properties of the coordinative variable during exercise may provide information about the dynamic mechanisms that lead to exhaustion.

## Introduction

Exhaustion is a ubiquitous phenomenon in physical activities, and it is particularly relevant in constantly performed tasks. Despite, however, the importance of understanding exhaustion for describing and predicting the limits of endurance, it has yet to be adequately modeled. Previous research, focused mainly on identifying and characterizing a specific site or process responsible for the exhaustion point, has generated a large body of results that, while important, are also controversial (Balagué et al., 2014a). Notably, reductionist approaches fail to consider the interaction dynamics that characterize complex psychobiological systems, ones capable of coordinating and reconfiguring their functions in order to fulfill continuously a task. Furthermore, exhaustion is a phenomenon manifested at the macroscopic level of action, and, therefore, it depends on a vast number of system component processes distributed across many levels and interacting over many time scales. Consequently, the study of coordinative or collective variables, which capture the dynamic products of interactions, would seem to offer an appropriate way of elucidating the mechanisms that lead to exhaustion during continuously performed motor tasks.

Due to the numerous terms and criteria used to describe exhaustion it is crucial to begin by clarifying some definitions of the term. The accumulation of effort is known to lead to the inability to maintain a predetermined task performance criterion, producing what is known as task failure (Gandevia, 2001). However, after task failure individuals who fulfill a predetermined task criterion are still able to continue the exertion at obviously lower intensity levels until total spontaneous task disengagement occurs. This spontaneous task disengagement, also known as the fatigue-induced spontaneous termination point (FISTP) (Hristovski and Balagué, 2010), is considered crucial for an adequate modeling of exhaustion.

Hristovski and Balagué (2010), using the elbow angle as a coordinative variable during a quasi-isometric exercise, showed how the FISTP was preceded by an enhancement of the elbow angle fluctuations on various time scales (from hundreds of milliseconds to tens of s). Because an intentional effort was needed to sustain the required elbow angle (90°) during the isometric contraction, the authors conclude that the enhancement of fluctuations reflects the impending instability of the neuromuscular axis, including the intentional processes of the performer.

In complex psychobiological systems the adaptability to task constraints is revealed by the emergence of synergies or coordinative structures forming action-perception cycles (Turvey, 1992). Synergies or equivalently coordinative structures (Kugler et al., 1980) have been defined as functional and context-sensitive units of biological action (Latash, 2008; Kelso, 2009). Components forming a particular synergy possess two characteristics: pleiotropy, i.e., capacity of same components to participate in different functional synergies (Glazier and Davids, 2009) and degeneracy, i.e., capacity of different components to be engaged in attaining the same goal (Edelman and Gally, 2001). The role of the components forming a motor synergy is their compensatory actions in preserving the task-goal performance of the psychobiological system.

In continuous motor tasks the control of a synergy is spread over some time interval. Hence, the compensatory effects of components of the system that form the synergy have to be also spread in time. Such adjustments to previous perturbations have to possess a temporal structure which does not allow the important performance variable, i.e., elbow angle, to make large fluctuations around the goal value but a structure which quickly reduces the fluctuations in any direction which deviates from the goal value. Quickly suppressed deviations, by applying counteracting actions would tend to produce smaller deviations around the goal value and thus a better, stabilizing, control. Hence, the temporal co-variation among the subsequent adjustments must be *negative* to enable the required constant task output by producing anti-persistent or sub-diffusive time variations. Otherwise, fluctuations of the component processes which would on average positively add to the already extant deviation would tend, especially on longer time scales, to produce larger deviations from the desired goal value, i.e., a destabilizing persistent or super-diffusive effect.

Such co-adaptive actions are applied by the neuro-musculo-skeletal system of performers by recruiting more/less motor units or/and engaging/disengaging energy transfer from other parts of body actions on the arm-bar system. These adjustments may be treated as components, i.e., elementary variables, of the synergy developing in time. Their temporal structure enables quicker or slower recovery of the local goal elbow-angle value.

Thus, compensatory movements and related adjustments are produced in order to intentionally stabilize any motor task during exercise. Specifically, in quasi-isometric exercises, these compensatory adjustments aim to correct any deviation from the task goal. Thus, the stabilizing control is reflected in the temporal structure and time variability properties of the coordinative variable under study.

Changes in the temporal variability of coordinative variables can be measured by analytical tools such as Detrended Fluctuation Analysis (DFA) (Delignières et al., 2011). DFA was first introduced by Peng et al. (1994) to determine long-correlations behavior in non-stationary time series. The output of this method is the exponent α, which provides information about the scaling properties of the signal. According to Eke et al. (2000), two general families of processes can be differentiated by the value of the α exponent: fractional Gaussian noises (fGn) and fractional Brownian motions (fBm). For 0.5 < α ≤ 1 the time series are generated with increments which are, on average, positively correlated in time, meaning that a positive trend in the past will more likely be followed by a positive trend in future; this is called persistent fGn. Its cumulative sum results in persistent or super-diffusive fBm with 1.5 < α < 2. For 1 < α < 1.5 an anti-persistent or sub-diffusive fBm process occurs in which increments are negatively correlated, that is, a positive trend in the past will more likely be followed by a negative trend in the future. The value of the exponent α is conceptually equivalent to other scaling exponent like Hurst's (H) exponent (Bassingthwaighte and Raymond, 1994).

There is some recent evidence to suggest that the effect of fatigue does reduce the structure of variability during some kinds of strenuous exercise (Pethick et al., 2015). For their part, Cashaback et al. (2013) observed a change in variability of the biceps brachii surface electromyography during an exhaustive submaximal isometric elbow flexion exercise. Hristovski et al. (2010) contended that the synergy stabilizing mode of perception-action would correspond to the anti-persistent structure of fluctuations of control in continuous motor tasks. Although isometric contractions exhibit greater stationarity in time series than do rhythmic contractions, the continuous adjustments (i.e., corrections in the coordinative variable) made during perception-action cycles and acting on the control of the movement may be of relevance when it comes to determining changes in the structure of variability.

In the present study we aimed to investigate the effect of time-on-task on fluctuations in the elbow angle (coordinative variable) during a quasi-isometric exercise performed until the FISTP. The main goals were to quantify changes in the temporal structure of fluctuation variability as the performer approaches the FISTP and to test the hypothesis of loss of psychobiological flexibility of motor control at the kinematic level of analysis (Balagué et al., 2014a). We hypothesized that for prolonged intervals on task, and close to the FISTP, the structure of the coordinative variable variability will undergo a shift toward Brownian motion as a consequence of the temporal relation shift in the inhibitory-excitatory processes relation along the psychobiological perception-action axis of performers. Were this hypothesis to be supported, it could lead to novel interpretations of the mechanisms that produce exhaustion in endurance motor tasks.

## Methods

### Participants

Seven Caucasian physical education students (males; *M* = 22.34 years old, *SD* = 3.47) with no sport specialization but engaged in a wide range of aerobic activities at least three times a week volunteered to participate in this study. Prior to taking part, they completed a questionnaire to confirm their health status, as well as an informed consent form, which was approved by the Clinical Research Ethics Committee of the Sports Administration of Catalonia.

### Procedure

**Figure 1:**
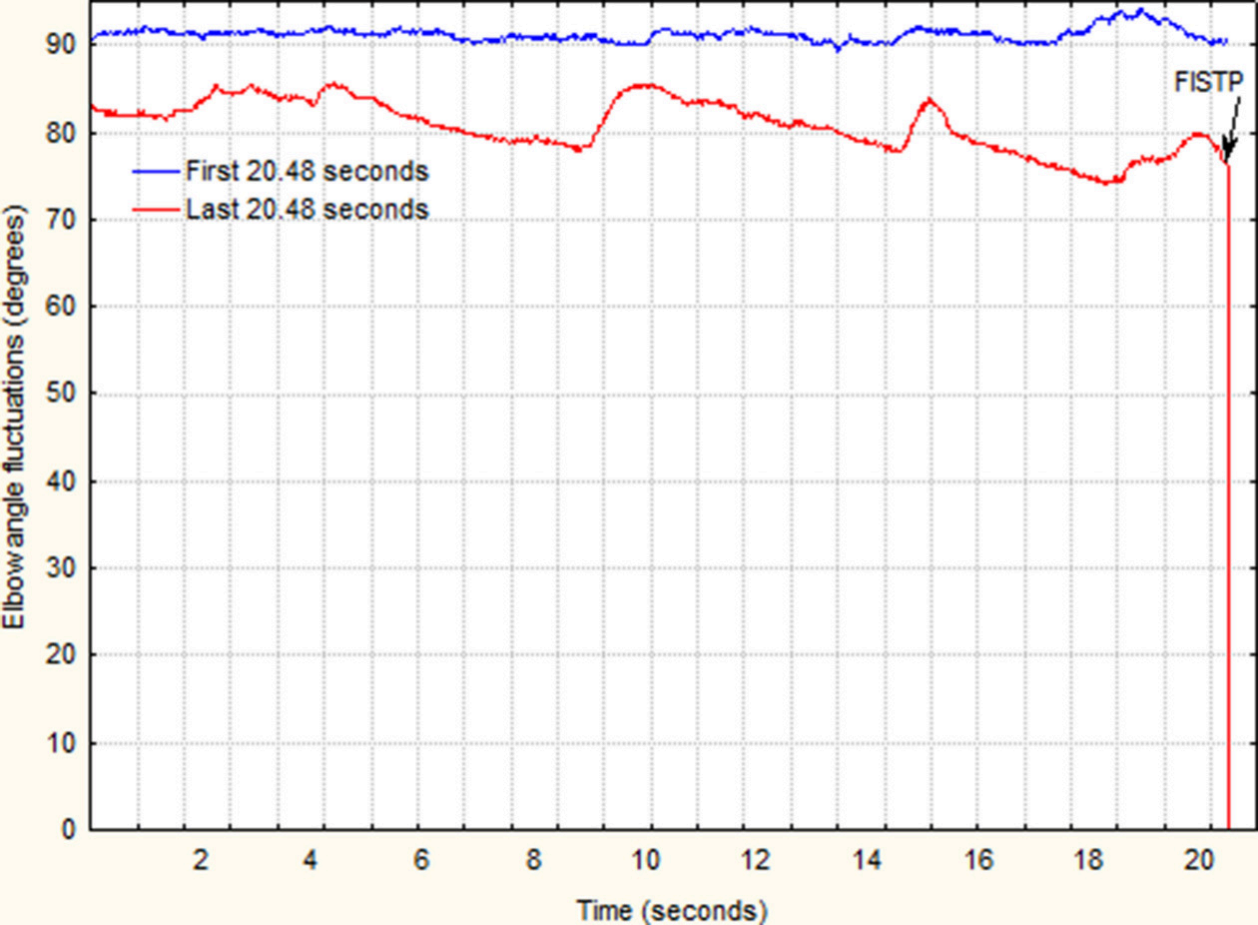
**Example of initial and final elbow angle fluctuations**.

On three different days over a period of 4 weeks, participants performed a quasi– isometric test holding an Olympic bar with an elbow flexion of 90° until the FISTP. (Hristovski and Balagué, 2010). Accordingly, they were asked to maintain intentionally a 90° angle at their elbow joint and to persist as close as possible to it even if the initial elbow angle was lost. One week prior to the test the weight of the bar for each participant was determined as 80% of the one-repetition maximum test (1RM) using an arm-curl exercise. During the trials participants had to sit on an inclined-forward bench in order to prevent possible spinal injuries. A reference cord was placed at the level of the participant's wrist in order to facilitate haptic and visual feedback on the initial position and its loss. To make the task more representative to real-world situations we did not fixed the elbows of participants but they were left to freely vary in all three dimensions. Before starting the test all participants were adjusted to the required position of 90° elbow flexion, with their wrists touching the reference cord. As shown in Figure 1 the trial was finished when the participant left the bar on its support (i.e., when the FISTP was reached). Variation in the elbow angle during the trials was recorded using an electrogoniometer (Biometrics) and its associated software (Ebiom). The sensors of the electrogoniometer were placed on marked points on the upper arm and forearm for both extremities and were adjusted to the required starting flexion of 90°. The sampling frequency was set at 50 Hz and the amplitude resolution was 0.1° for each extremity.

### Data analysis

To study changes in the time series structure we first determined the FISTP as an abrupt and persistent switch toward negative values in the differentiated time series calculated as y = x-x(lag = 1) where x denotes the elbow angle, x(lag = 1) the lagged elbow angle for 1 data point and y denotes the angle change (Hristovski and Balagué, 2010). The mean length of the time series was 5878 data points; *SD* = 1417 data points. Figure 1 shows an example of the initial and final periods of the elbow angle fluctuations. The mean of data points until the FISTP was 5703 data points with *SD* = 1409. A total of 84 time series were analyzed: 7 (participants) × 2 (arms) × 3 (trials) × 2 (time on task periods). The time series analyzed are available as Supplementary Material. Changes in the structure of the variability in each time series were calculated by using the DFA:

#### Detrended fluctuation analysis (DFA)

According to Ihlen's code for Matlab® (Ihlen, 2012). To illustrate briefly the DFA analysis, the total length of elbow angle fluctuations (N) was first integrated by Equation (1).

(1) $$\begin{array}{l} Y\left(i\right)\equiv \overset{i}{\underset{k=1}{\sum }}\left[x_{k}-\;\langle x\rangle \right] \end{array}$$

Where x_k_ is the elbow fluctuation signal and 〈x〉 is the average elbow fluctuation of the *N* samples. A polynomial function of order 2 was then used to fit the time series in order to calculate the local trend (Ihlen, 2012). The resulting time series were divided into different boxes *n* of equal length (i.e., scales), with the local trend being subtracted in each box. Because the DFA works with power of 2 sample data points the largest time scale that was analyzed contained 1024 data points. For each box the root mean square (RMS) fluctuation was calculated by using Equation (2).

(2) $$\begin{array}{l} RMS=\sqrt{\frac{1}{N}\overset{N}{\underset{k=1}{\sum }}{\left[y\left(k\right)-y_{n}\left(k\right)\right]}^{2}} \end{array}$$

**Figure 2:**
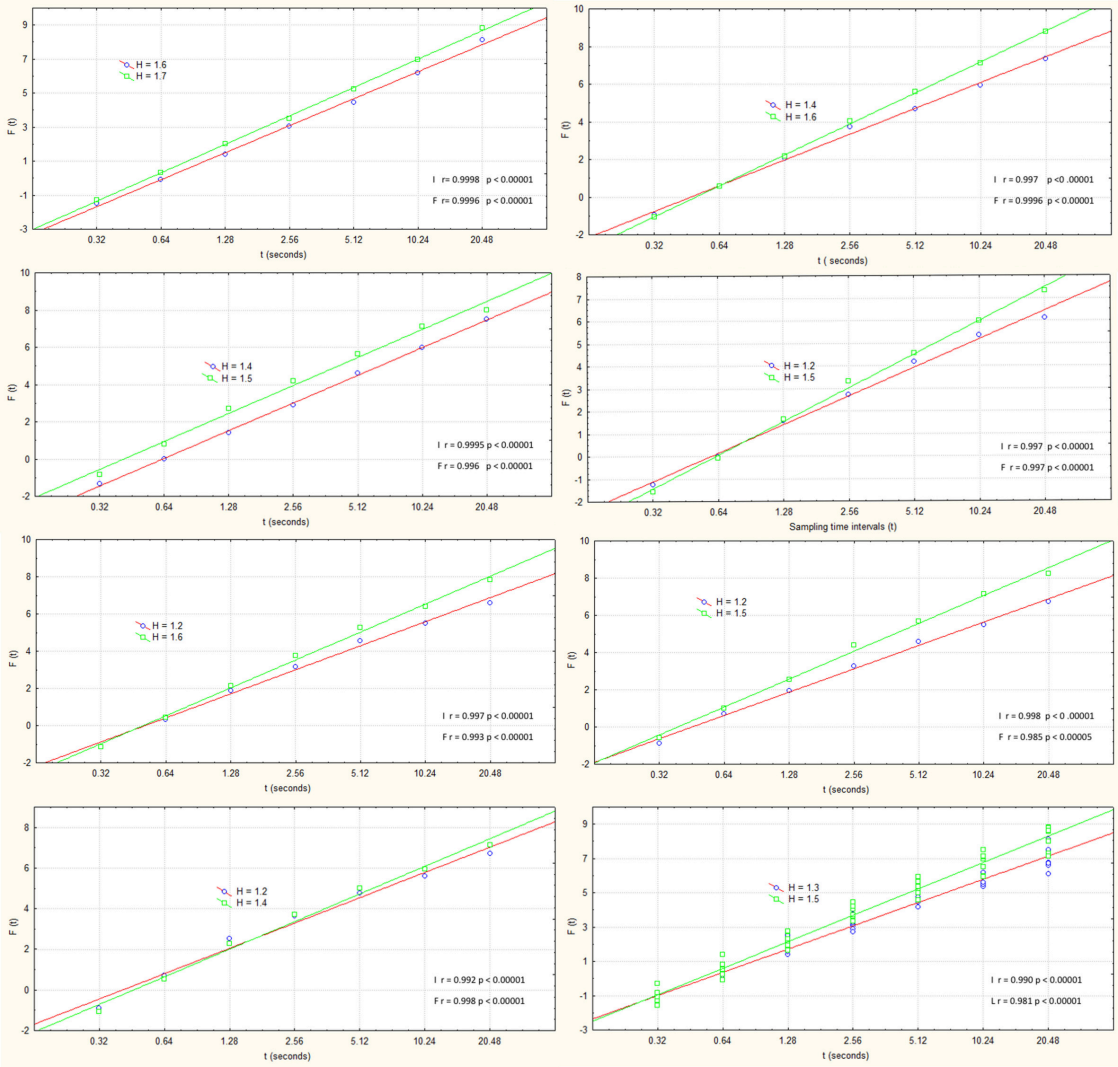
**Regression curves and H exponents for the seven participants in the initial (red line) and final (green line) periods of exercise pooled over both arms**. In the lower right corner is a regression curve pooled over all participants. r—correlation of linear fit to data; p-significance level. I—initial and F—final time series. Horizontal axis: Time window of sampling; Vertical axis: Fluctuation size is given as log2 RMS.

Where the *y*(*k*) are the integrated time series and the *yn(k)* is the local trend in each box. The H exponent, obtained as the slope value of the regression line between the scale and local fluctuations on a log-log diffusion plot, was used to determine the structure of the time series fluctuations (see Figure 2).

#### Statistics

Effects of [trial (3) × arm (2) × time on task (2)] on the Hurst exponent values were analyzed by a Three-way fixed design RM ANOVA. Also effects of [Trial (3) × Arm (2) on Time on task (1)] for the initial time series as well as [Trial (3) × Arm (2) on Time on task (1)] for the final time series Hurst exponents was conducted by a Two-way RM ANOVA. The same design was applied for checking effects of [Sampling timescale (7) × Time on Task (2)]. To test for pairwise differences in comparisons where main effects attained significant values Tukey HSD *post-hoc* comparison tests were applied. We additionally performed a correlation analysis between the elbow angle fluctuations of both arms to check for possible shared variance of these sub-systems.

## Results

All participants accomplished successfully the requirements of the experiment, reaching the FISTP on average in 122.2 s (*SD* = 29.2).

### RM ANOVA

**Figure 3:**
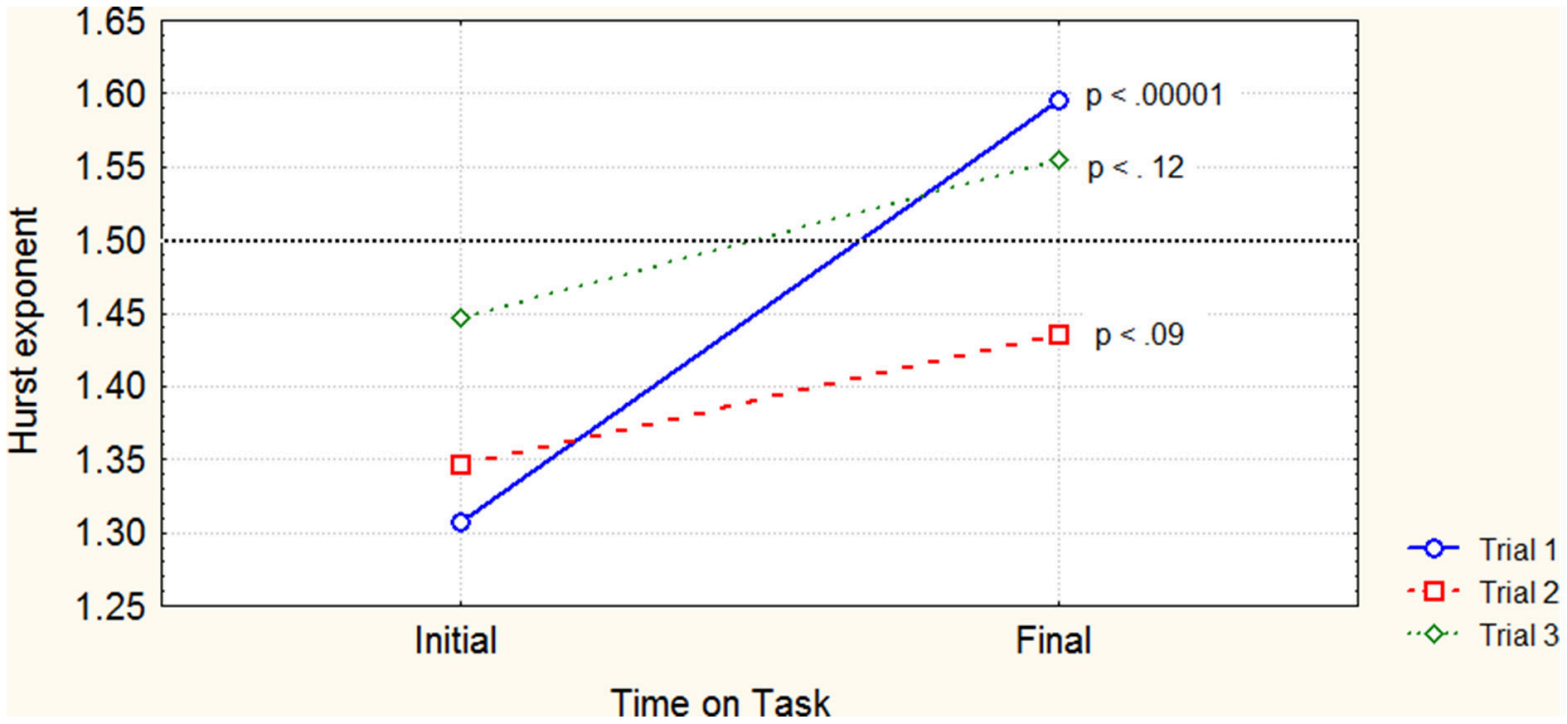
**Trial vs. Time on task interaction with associated significance values of mean Hurst exponent differences**. The dotted line marks the border between anti-persistent (sub-diffusive) and persistent (super-diffusive) Hurst values.

**Figure 4:**
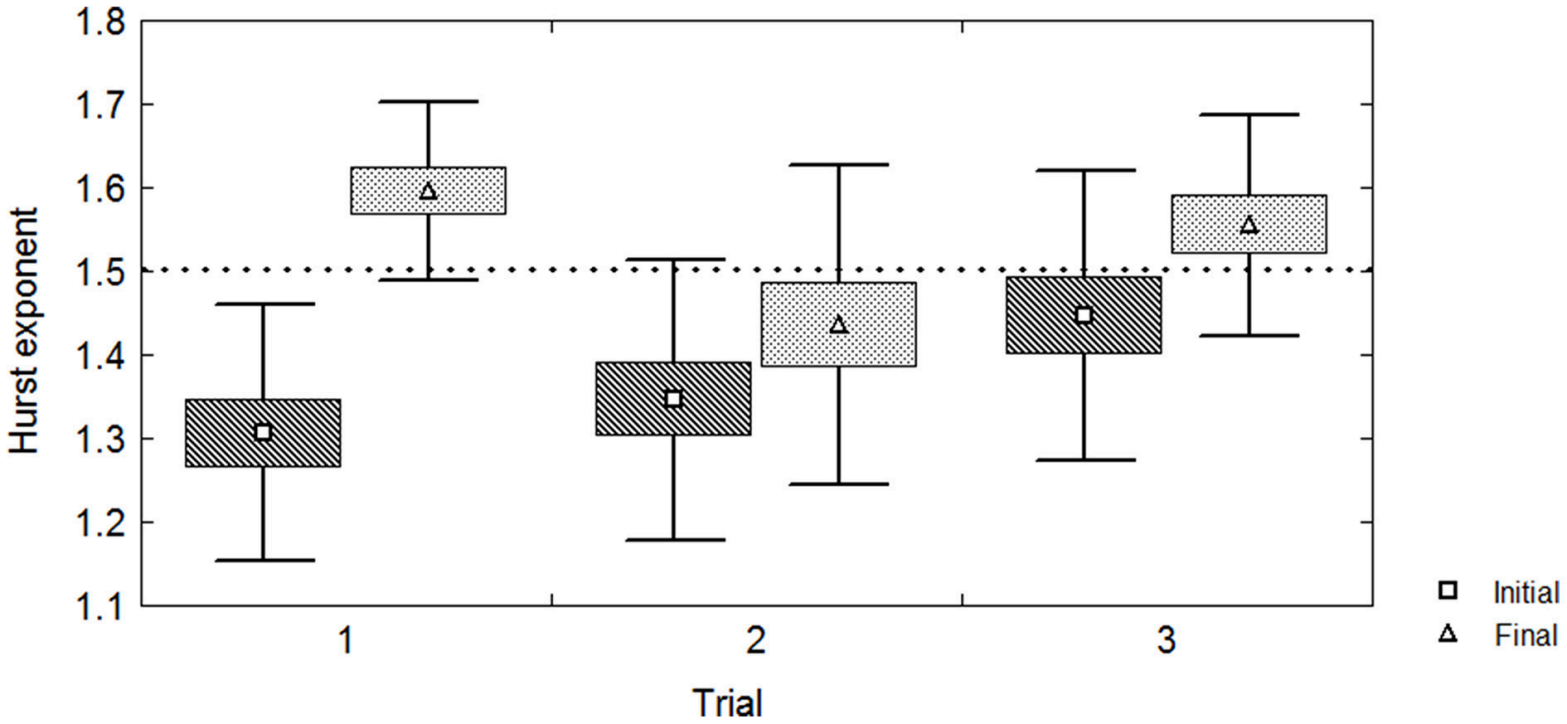
**Pair-wise comparisons of Hurst exponent (initial and final) differences vs. Trial number**. The dotted line marks the border between anti-persistent (sub-diffusive) and persistent (super-diffusive) Hurst values.

The three-way fixed effects RM ANOVA revealed a main effect of time on task on the Hurst exponent value *F*_(1, 36)_ = 29.62; *p* < 0.00001. There were no significant effects of the Trial *F*_(2, 36)_ = 2.65; *p* < 0.085; and Arm *F*_(1, 36)_ = 0.23; *p* < 0.633 on the mean Hurst exponent values respectively. It also revealed a significant interaction Trial (3) × Time on task (2) *F*_(2, 36)_ = 4.30; *p* < 0.02 (Figure 3). However, there were no significant three-way [Trial (3) × Arm (2)] × Time on task (2) interactions *F*_(2, 36)_ = 0.10; *p* < 0.91, as well as Two-way [Trial (3) × Arm (2)] *F*_(2, 36)_ = 0.48; *p* < 0.62, nor [Arm (2) × Time on task (2)] *F*_(1, 36)_ = 0.31; *p* < 0.58 interactions. *Post-hoc* comparisons showed a significant difference between the mean Hurst exponent of initial and final period for Trial 1 *p* < 0.00001, but showed no significant differences for Trial 2 *p* < 0.09 and Trial 3 *p* < 0.12 (Figures 3, 4).

The Two-way RM ANOVA for [Trial (3) on Time on task (1)] for the initial time series revealed no significant main effect of trials on the Hurst exponent *F*_(2, 36)_ = 2.55; *p* < 0.0923. However, the [Trial (3) on Time on task (1)] revealed a significant main effect *F*_(2, 36)_ = 4.20; *p* < 0.0230 for the final time series. *Post-hoc* comparisons revealed a significant difference between the mean Hurst exponents belonging to the first and the second trial *p* < 0.022.

**Figure 5:**
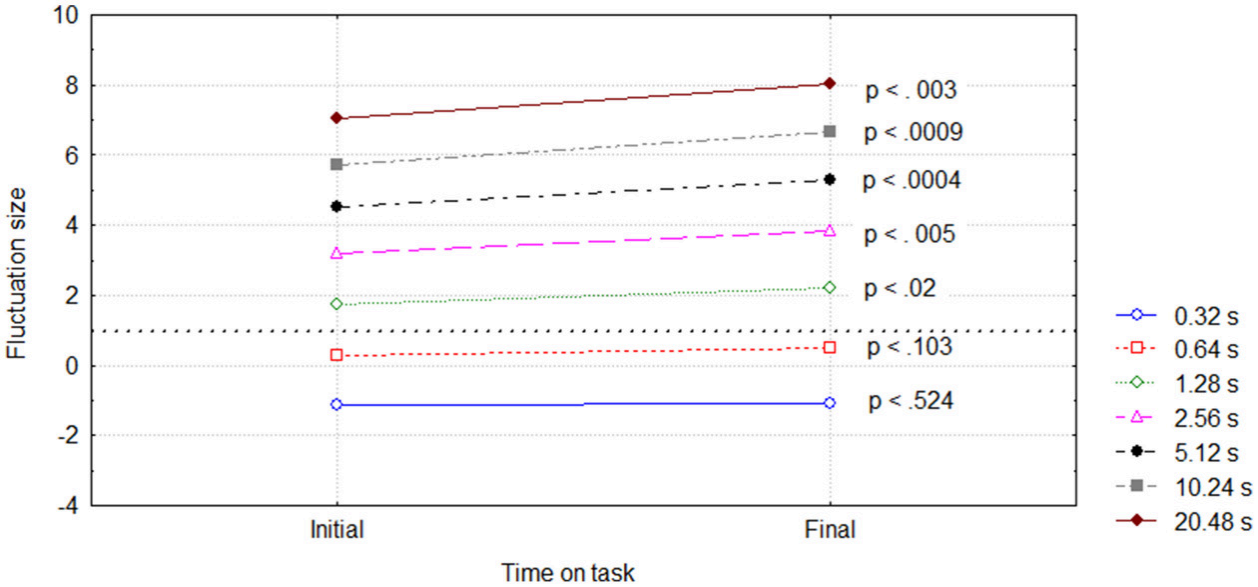
**Interaction of the Sampling timescale (right handside legend) and Time on task (horizontal axis) on the Fluctuation size (given as log_**2**_ RMS)**. The dotted line shows the timescale on which the fluctuation size of final period time series become significantly larger in comparison with initial period time series.

The Two-way RM ANOVA for [Sampling timescale (7) × Time on Task (2)] revealed significant main effect *F*_(1, 42)_ = 113.06; *p* < 0.00001 on the fluctuation size differences between initial and final periods, as well as significant two-way interaction *F*_(6, 42)_ = 5.57; *p* < 0.0003 (Figure 5).

### Between arms correlations of hurst exponents

Significant Pearson correlation coefficients between the right and left arm elbow angle H values were obtained for the initial (*r* = 0.772; *p* < 0.0001) and the final time on task (*r* = 0.691; *p* < 0.0005), but there was no significant difference between correlation coefficients (*z* = 0.530; *p* < 0.596).

## Discussion

The main aim of this research was to help clarify the dynamic mechanisms that lead to exhaustion at the action level by extending some preliminary results regarding motor control in time continuous tasks under developing fatigue (Hristovski et al., 2010; Balagué et al., 2014b). In applying the notion of task goal stabilizing synergy (Latash, 2008; Kelso, 2009) to processes spread across time scales we hypothesized that: under the non-fatigue condition it would have to be manifested as a dominantly anti-persistent process. This process would be detectable by time series analytic techniques such as DFA. Contrary to this, when the goal directed synergy is subject to accumulated fatigue then the control of the elbow angle would tend to be of a persistent type. We hypothesized that this is due to the impaired ability of the psychobiological system to make short-term adjustments.

Multi-way RM ANOVA results of the initial period of the task revealed a consistent tendency toward anti-persistent fBm of the elbow angle in both limbs across all three trials. The timescale on which on average the fluctuations in the final part close to FISTP became significantly larger than those in the initial part was close to 1 s. On all timescales larger than this up to the largest one with period of 20.48 s the fluctuations tend to be larger in the final part of the exercise. In almost all 84 cases, however, this was followed in the final period by a change in the slopes of the DFA plot of the fluctuations, revealing a tendency toward persistent fBm. These observations corroborate the hypothesis that a goal stabilizing synergy that is spread over longer time periods in a non-fatigued system tends to be of anti-persistent type. On the one hand, the anti-persistent fBm is a non-stationary process which serves to regulate the force production around the goal value, but since it is anti-correlated it confines the system close to it. On the other hand, a persistent fBm is a poor strategy for maintaining constant task goals because it creates an overly itinerant behavior which does not attain the task goal if not reduced with time. This shift from anti-persistent to persistent fBm possibly reflects that the dynamics within the system becomes increasingly low dimensional, and that the cooperative and competitive behavior of component processes dwell on longer time intervals. In the initial period of the task the temporal sequences of weak fluctuations signify a potential for more flexible control of the goal variable. By contrast, in the final period the enhanced long-period fluctuations possibly signify an increasingly coherent and, therefore, less flexible control of the ongoing activity. These increasingly coherent yet competitive processes seem to be responsible for the enhanced low-frequency variability close to the FISTP. The significant shared variance of H exponents between both arms, as revealed by the correlation analysis, points to the integrated control by both arms. However, the substantial portion of variance that was not shared bilaterally shows also the existence of autonomy in the control of each arm separately (Kelso, 2012). From a kinematic point of view, the anti-persistent dynamics of the elbow angle consist of weak fluctuations around the goal value of 90°, due to the fine adjustments made. These fine adjustments are psycho-physiologically produced by the intentionally sustained cooperation among the higher control loops (presumably responsible for task specific perception, attention, motivation), down to spinal reflexes and muscular processes which vary on much shorter time scales. It is important to bear in mind that for anti-persistent processes the subsequent increments in fluctuations are negatively correlated, satisfying the definition of a goal stabilizing synergy. The balance of excitation and inhibition is a general mechanism for stabilizing the activity level in neural tissue (Shu et al., 2003; Higley and Contreras, 2006; Shew et al., 2009), and it has also been demonstrated in computational models of neural networks (van Vreeswijk and Sompolinsky, 1996; Brunel, 2000). That said, any positive fluctuation, as a consequence of central excitation, is on average compensated for by a subsequent negative fluctuation that arises as a result of joint coupling between the central inhibition and the pull of gravity. These kinds of negative feedback loops are well-known mechanisms for generating anti-persistent time series (Cuomo et al., 2000). From the results obtained in this research, the anti-persistent type of feedback was possibly implemented by having a greater available number of degrees of freedom, such that a more fine-grained time-amplitude control of the goal variable could be achieved. In other words, the inhibition-excitation control processes were competing on short time scales. For kinematic fluctuations in isometric exercises, this would mean that different articulatory degrees of freedom are served by different but anti-correlated neural networks. In order to account for the DFA results, networks must be initially sufficiently distinct such that neural activity in one network is only weakly coupled to activity in other networks (e.g., only partially overlapping). At the beginning of the exercise these anti-correlated distinct networks are able to provide more independent and, therefore, fine-grained time-amplitude control of the goal coordination variable. Through competition on short time scales (e.g., unconscious reflexes), the anti-persistent fBm is characterized by relatively balanced high to low frequency fluctuation amplitudes. As the task continues and fatigue develops, however, this fine-grained control, based on short-time competition, loses its grip and the competition shifts toward longer term intervals. In our opinion this effect is underpinned by the protective behavioral inhibition, consisting of processes that encompass the neural and muscular changes (e.g., neuromuscular tension; Gandevia, 2001) that result in a delayed possibility of adjustment of the goal variable. The metabolic inhibition might be reflected, for example, by the lower contractile ability of some portion of muscle fibers and provoke a greater inhibition effect.

The increased neurotransmitter levels in some central nervous system synapses (Cotel et al., 2013) may also heighten the inhibitory effects at this level. The accumulated effort enhances the growth of this phase and it becomes increasingly macroscopic. As fatigue, i.e., neuro-metabolic inhibition sets in these networks start to perform more in unison, with the excitatory influence being constrained by longer-term motivational loops. To be able to adjust the goal variable, a highly motivated performer has to intentionally activate an increasingly larger population of motor units to compensate the inhibitory influences.

The competition of these growing coalitions then begins to unfold over longer time scales. In other words, the control becomes coarser with respect to time. Finely-grained, high frequency-low amplitude modes contributions decrease in comparison to the coarse-grained, low frequency-high amplitude modes adjustments generated dominantly by long-term varying attention-motivation loops. The dominant portion of control is thus transferred to conscious, intentional, long-term, coarser grained loops. This means that the motivationally supported excitatory phase also segregates, becoming more coherent in order to counteract the increasing inhibition. Thus, what emerges is a formation of two macroscopic competing coalitions. Because intention-motivation control loops function on longer time scales (Kiebel et al., 2008) than do spinal and subcortical control loops, the adjustment delay shifts to longer time intervals. The competition between the intention to sustain the task and the progressive loss of neuromuscular tension is illustrated by the amplitude increase of long-term fluctuations in goal variable values during the final period of the exercise. This enhancement of low-frequency elbow angle fluctuations precedes the abrupt reduction in the angle which coincides with the FISTP, as manifested by the tendency toward larger H values. Similar results were obtained previously on the basis of analysis of other measures such as SD and the power spectrum profiles of elbow angle fluctuations in participants performing the same task (Hristovski and Balagué, 2010). However, the obtained trial vs. time on task interaction effects pointed to a more involved picture of these processes. There is apparently a long-term, i.e., a week to week, variability of the temporal structure of quasi-isometric control. Although in all three trials there was a tendency of participants to initially engage in an anti-persistent regime of control and in the final part to approach the persistent regime, this effect was most clear only on the first trial. Changes in attention mood, motivation or change in control strategy by engaging/disengaging energy transfer from other parts of body on the resistance system over period of weeks may be responsible for this kind of a nested temporal process. However, from this research we are not able to discuss in more detail about the factors which may be responsible for this variation.

In addition, two main potential methodological limitations should be mentioned here: the scarce number of participants and the lack of taking into account for analysis other biomechanical degrees of freedom of the shoulder and elbow joint system. The later may account for the different strategies that participants used to negotiate the fatigue. In this research, we used only DFA method because we were specifically interested in the time variability structure of quasi-isometric exercises as a supplement to previous research findings. These findings used other measures of variability such as the SD and the power-spectrum shift of elbow-angle fluctuations (Hristovski and Balagué, 2010). The behaviors of both measures were consistent with present findings concerning the enhancement of low-frequency fluctuations with developing fatigue and close to FISTP.

In summary, the path to task disengagement seems to involve, on the coordination level, temporally nested psychobiological changes. At larger time scales, i.e., weeks, there seems to exist a slow process of variation possibly affected by change in personal constraints and in connection to negotiating the fatigue that develops on much shorter timescales during a sole exercise bout. At this timescale there exists a tendency toward a gradual shift from weakly coupled, short-term, anti-correlated activity toward more-strongly coupled, long-term correlated activity of competing psychobiological networks. The final task disengagement event (FISTP) in this scenario may be considered as a giant, i.e., coherent, protective inhibitory fluctuation that causes a temporary abrupt switch to the low energy expenditure level. More generally, this mechanism may be considered as an evolutionary stabilized protective decision-making process. There would appear to be a compelling case to link this understanding of how fatigue manifests on the coordination level with percolation processes and the formation of giant components (Breskin et al., 2006) that are prominent in complex systems, such as human psychobiological networks. In future research it is warranted to examine the viability of this hypothesis on both the experimental and modeling levels so as to contribute further to an understanding of the consequences of exercise-induced fatigue and the identification of the limits of endurance.

## Author contributions

PV Conception and design of the experiment. Analysis and interpretation of data. Drafting or revising the article. RH Conception and design of the experiment. Analysis and interpretation of data. Drafting or revising the article. NB Conception. Interpretation of the data. Drafting or revising the article. The authors approved the final version and agree to be accountable for all aspects of the work.

## Funding

This study was supported by the Institut Nacional d'Educació Física de Catalunya (INEFC), the Generalitat de Catalunya.

### Conflict of interest statement

The authors declare that the research was conducted in the absence of any commercial or financial relationships that could be construed as a potential conflict of interest.
